# Natural succession and clearcutting as drivers of environmental heterogeneity and beta diversity in North American boreal forests

**DOI:** 10.1371/journal.pone.0206931

**Published:** 2018-11-02

**Authors:** Sergio García-Tejero, John R. Spence, John O’Halloran, Stephane Bourassa, Anne Oxbrough

**Affiliations:** 1 Department of Biology, Edge Hill University, Lancashire, United Kingdom; 2 Department of Renewable Resources, University of Alberta, Edmonton, Canada; 3 School of Biology, Earth and Environmental Sciences, University College Cork, Cork, Ireland; 4 Laurentian Forestry Centre, Québec, Canada; Chinese Academy of Forestry, CHINA

## Abstract

Clear-cutting alters natural ecosystem processes by reducing landscape heterogeneity. It is the dominant harvesting technique across the boreal zone, yet understanding of how environmental heterogeneity and beta diversity are structured in forest ecosystems and post-clear cut is lacking. We use ground-dwelling arthropods as models to determine how natural succession (progression from deciduous to mixed to coniferous cover types) and clear-cutting change boreal forests, exploring the role of environmental heterogeneity in shaping beta diversity across multiple spatial scales (*between-cover types* and *between-stands of the same cover type* (1600 to 8500 m), *between-plots* (100 to 400 m) and *within-plots* (20 to 40 m)). We characterise environmental heterogeneity as variability in combined structural, vegetational and soil parameters, and beta diversity, as variability in assemblage composition. Clear-cutting homogenised forest environments across all spatial scales, reducing total environmental heterogeneity by 35%. Arthropod beta diversity reflected these changes at larger scales suggesting that environmental heterogeneity is useful in explaining beta diversity both *between-cover types* and *between-stands* of the same cover type. However, at smaller scales, *within-* and *between-plots* spider beta diversity reflected the lower environmental heterogeneity in regenerating stands, whereas staphylinid and carabids assemblages were not homogenised 12 years post-harvest. Differences in environmental heterogeneity and staphylinid beta diversity between cover types were also important at small scales. In regenerating stands, we detected a subtle yet notable effect of pre-felling cover type on environmental heterogeneity and arthropods, where pre-felling cover type accounted for a significant amount of variance in beta diversity, indicating that biological legacies (e.g. soil pH reflecting pre-harvest conditions) may have a role in driving beta diversity even 12 years post-harvest. This study highlights the importance of understanding site history when predicting impacts of change in forest ecosystems. Further, to understand drivers of beta diversity we must identify biological legacies shaping community structure.

## Introduction

Sustainable forest management seeks to conserve ecological processes and biodiversity across forested landscapes [1,2]. The approach of natural disturbance emulation aims to achieve this through knowledge of natural forest dynamics and heterogeneity at multiple spatial and temporal scales [2,3], as well as rigorous assessment of how forest management effects ecosystem components [4].

Boreal mixedwood forests are heterogeneous across landscapes; a shifting mosaic of stands in various shapes, sizes and stages of succession. Natural succession begins with a disturbance (e.g., fire or wind) that creates gaps and facilitates establishment of shade-intolerant deciduous saplings. As succession proceeds and deciduous trees grow, coniferous saplings establish in the shaded conditions, and a multi-layered mixed canopy develops, enhancing structural complexity and the range of habitats for understory biota, thus fostering their diversity [4]. Eventually, conifers dominate, and later stages of succession may sustain higher species diversity due to resource specialisation and niche partitioning as classical ecological theories suggest [5, 6]. At smaller scales, within a stand, differences in tree species composition, age, size and spatial arrangement influence the distribution of light, water, carbon, nutrients and pH, which shape the understory and together determine microclimatic conditions and resource availability for forest-dwelling organisms [2, 7]. Thus, natural succession profoundly influences the degree of environmental heterogeneity across landscapes but also within stands.

Despite increasing interest in alternative felling methods that seek to minimise impacts to biodiversity and ecosystem function by emulating more natural conditions (e.g., retention harvest, continuous cover forestry), clear-cutting remains the dominant harvesting technique across the boreal zone [8, 9]. Clear-cutting alters natural ecosystem dynamics and results in a simplification of the environment across scales [4, 10], reducing landscape heterogeneity and smaller-scale structural complexity by replacing a variety of successional stages with homogenously aged stands. Further, clear-cuts differ from natural disturbances as they retain few biological legacies such as live or dead trees, or fallen woody debris, which are ‘keystone structures’ providing resources crucial for a wide range of species [11, 12].

For forest biota, the impacts of clear-cutting are relatively consistent across habitats and taxonomic groups, leading to a local reduction in alpha diversity [13]. In the boreal zone, this has been attributed to a decrease in old-growth associated species including bryophytes and lichens [14], vascular plants [15] and invertebrates [16, 17]. Beta diversity is an important component of gamma (or landscape) diversity, and hence the spatial structure of species populations within ecosystems. Further, to understand the impacts of human induced changes on biodiversity, we must have a proper knowledge of how beta diversity is structured before and after disturbance and of how it is created and maintained [2, 18]. Few studies have investigated the impact of clear cutting on beta diversity in boreal forests, with varied responses depending on the taxonomic group studied. [15] found the response of plant beta diversity two years post-harvest depends on both forest type and spatial scale explored, whereas [19] found pollinator beta diversity was enhanced 12 years post-harvest, likely reflecting the varied nature of the open habitats created. Despite this recent work, the role of environmental heterogeneity in shaping beta diversity across spatial scales following clear cut remains largely unexplored.

Here, we use ground-dwelling arthropods (Araneae; Coleoptera: Staphylinidae and Carabidae) as models to address this knowledge gap in the context of boreal forest management. Ground dwelling arthropods are diverse and abundant in boreal mixedwoods [7] and their assemblages are strongly influenced by environmental change in forest ecosystems [17, 20, 21]. Using information from several taxonomic groups incorporates responses of various trophic levels and ecological niches, which may be differently affected by environmental change. Spiders are predatory and predominately influenced by habitat structure and prey availability [22], staphylinids are mainly predators, saprophagous or mycophagous [23], whereas most carabids are generalist predators, but a minority are seed-eaters or omnivores [24]. Given their small size and the patchiness of the resources on which they depend, ground-dwelling arthropods respond to environmental change at a much finer scale than usually considered in forest management. As such, they may respond to homogenisation associated with clear cuts across a wide range of spatial scales.

We determine how environmental heterogeneity and arthropod beta diversity are structured across spatial scales (1600 to 8500 m; 100 to 400 m; 20 to 40 m) in boreal mixedwoods. We ask how they are affected by clear-cutting (comparing mature stands with 12-year post-harvest) and stage of succession (early—deciduous dominated; mid—mixed stands of deciduous and conifer; late -conifer dominated). Specifically, we address:

- How are environmental heterogeneity and beta diversity affected by stand development and clear-cutting within stands?
- How much does spatial scale and forest cover type contribute to environmental heterogeneity and arthropod beta diversity in mature and regenerating forests?
- What environmental characteristics influence arthropod beta diversity and what is their importance at each spatial scale?

## Methods

### Experimental design and data collection

**Fig 1:**
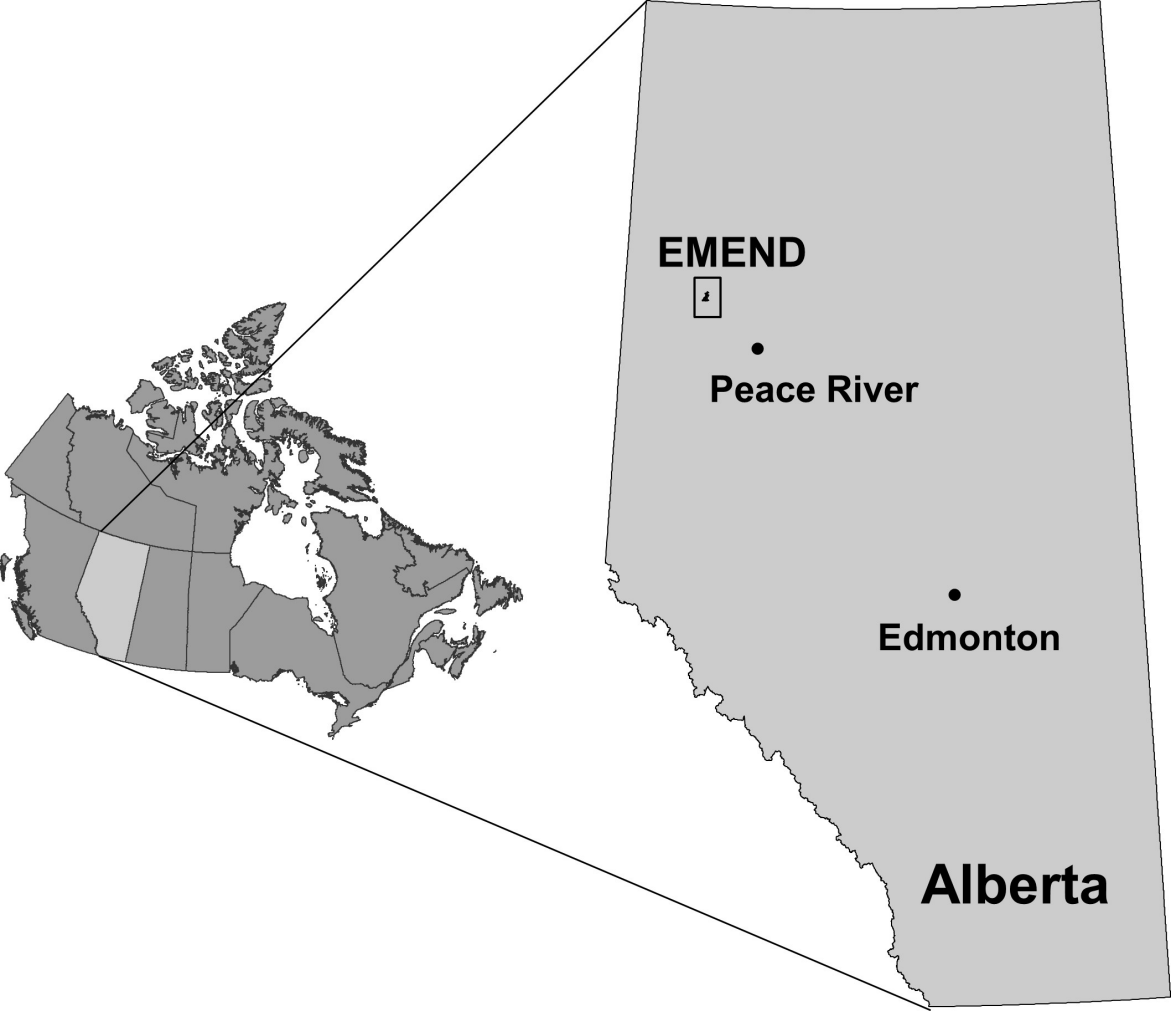
Location of sampling area at the EMEND research project in Northern Alberta, Canada.

Our study was undertaken at EMEND (Ecosystem Management Emulating Natural Disturbance), a long-term (est. 1998) research project of 100 stands exploring impact of variable retention harvest on ecosystem integrity. Located in the mixedwood boreal forests of northwest Alberta, Canada (56° 46’ 13” N– 118° 22’ 28” W) (Fig 1), the EMEND site is in Lower Boreal-Cordilleran Ecoregion, at an elevation of 677 m– 880 m. The region has an average annual precipitation of 387mm, with average annual maximum and minimum temperatures of 7.3° C and -4.2°C. We selected a subset of the EMEND experimental stands representing the three main canopy cover types in the region along a successional gradient of young to old following the description in [25]. These were: deciduous-dominated, which comprised >70% cover of deciduous trees (*Populus tremuloides* Michaux, *P*. *balsamifera* L., *Betula papyrifera* Marshall); mixed, which had a canopy cover of both deciduous and conifer species at 40–60% each *(Picea glauca (Moench) Voss*, *P*. *mariana (Mill*.*) Britton*, *Sterns* & Poggenburg, *Abies balsamea* (L.) Mill.); and, conifer-dominated, with >70% cover of conifer trees. For each of these forest cover types two development stages were sampled: ‘mature’, including trees ≥100 years old and developed to a level for commercial harvesting; and, ‘regenerating’, comprising naturally regenerating deciduous stands 12 years post-harvest. Pre-harvest basal area of these stands ranged from 33–38 m^2^ ha^-1^ and they previously comprised mature trees of the three developmental stages described above. Harvesting was done to industry norms of clear-cutting with 98–99% stem removal using feller bunching and direct route skidding. Thus, remaining above ground structures comprised randomly dispersed stems (1–2% uncut), stumps of mature cut trees and some brash on the ground. No post-harvest herbicide was applied. For more details on the experimental set up see [15] and references therein.

Three c. 10-ha stands of each cover type x development stage combination were sampled giving a total of 18 stands. The largest scale was derived from stands which were on average 5339 m apart (range 1646–8499 m). At this scale, two comparisons were defined within each development stage: *between-cover types* which was derived from the three stands of the same forest cover type combined (the pre-harvest cover type in the case of regenerating stands), and, *between-stands* derived from the three stands of the same forest cover type. In each stand, the *between-plots* scale was derived from three sampling plots, established in areas representative of the stand, which on average were separated 192 m from each other (100–400 m) and at least 50 m from the stand edge. In each plot, the smallest *within-plots* scale was derived from three sampling points that were established in a row, separated by 20–40 m. For ground-dwelling arthropods in forests this distance is sufficient to sample different individuals and hence infer statistical independence [26].

At each sampling point, one pitfall trap 11 cm in diameter was inserted into the ground, flush to the soil surface. These collect epigeal arthropods, and do not give an absolute measure of abundance but instead relative density of ground-active arthropods. Pitfalls are the most commonly used method for sampling ground-active arthropods in terrestrial environments [27] collecting high numbers of individuals for low effort and cost. Pitfall traps were partially filled with silicate-free ethylene glycol to serve as a killing agent and preservative. Traps were emptied once every three weeks over 12 weeks from late May 2010, after snow melt. This encompasses the main arthropod activity period in the region. Arthropod samples were sorted and adult spiders, carabid and staphylinid beetles were identified to species using available keys (S1 Table). Staphylinids in the subfamily Aleocharinae and the genus *Omalium* could not be reliably identified to species and were excluded from analyses.

To characterise the stands we measured tree height, diameter at breast height and stem density in each sampling plot. In mature stands measurements were taken in a 10 x 10 m area with the middle sampling point (pitfall trap) located at the centre, whereas in regenerating stands measurements were taken in two 2 x 2 m areas located 2m either side of the central pitfall trap in the direction of the outer traps. The sampled area was smaller in regenerating stands reflecting the much higher density of stems.

To determine environmental heterogeneity we measured variables that could influence arthropod species composition at each sampling point (Table 1). Soil pH and the depth of organic soil were obtained from a soil core sample. Organic soil depth was measured separately in three layers at each sampling point following [28]: litter, the uppermost layer where vegetation structures are recognisable; fermentation, the middle layer of partly decomposed organic material; and humus, the lowest layer of organic material. Percentage cover of litter, deadwood and vegetation were estimated in one square-metre quadrat, located at each sampling point, using the following classifications: litter (combining dead grass/thatch, deciduous leaves and needles); fine woody debris; coarse woody debris; ground vegetation (<10 cm high), distinguished into bryophytes and vascular plants; lower vegetation (10–50 cm high), distinguished into grass, other vascular plants and woody plants; upper vegetation (50–200 cm high). Percentage canopy openness was estimated using a spherical densiometer at each sampling point. Further, the number of stems of each tree species were counted to calculate the proportion of conifers and estimate tree species diversity using the exponential of Shannon-Wiener index in a 10x10m area at each sampling point, where the pitfall trap was the centre.

**Table 1:** Environmental characteristics (mean ± SE) that differ significantly by cover type (deciduous-dominated (DD), mixed (MX) and conifer-dominated (CD) stands), stage (mature and regenerating forests) and their interaction. Variables with no significant difference can be found in S5 Table.

	Mature			Regenerating			Permutational ANOVA		
	DD	MX	CD	DD	MX	CD	Cover type	Stage	Interaction
Median tree height (m) †	18.24 ± 1.30a	23.62 ± 1.67a	22.43 ± 1.54a	4.92 ± 0.22b	4.46 ± 0.21b	5.43 ± 0.19b	n.s	*F*_1,48_ = 108.0***	n.s
Median DBH (cm) †	19.24 ± 1.56a	24.05 ± 2.03a	25.43 ± 2.56a	2.99 ± 0.10b	2.95 ± 0.11b	3.24 ± 0.11b	n.s	*F*_1,48_ = 83.2***	n.s
Stem density (stems/m^2^) †	0.11 ± 0.01b	0.10 ± 0.01b	0.08 ± 0.01b	3.29 ± 0.23a	2.13 ± 0.16a	2.79 ± 0.24a	n.s	*F*_1,48_ = 139.4***	n.s
Soil pH	5.27 ± 0.09a	4.32 ± 0.08b	4.89 ± 0.16ab	5.42 ± 0.10a	4.82 ± 0.11b	4.90 ± 0.11ab	*F*_2,156_ = 26.2*	n.s	n.s
Litter soil layer depth (cm)	0.82 ± 0.10ab	1.51 ± 0.25ab	2.12 ± 0.19a	0.98 ± 0.09b	0.97 ± 0.11b	0.90 ± 0.08b	n.s	*F*_1,156_ = 19.6*	*F*_2,156_ = 10.9*
Fermentation soil layer depth (cm)	1.51 ± 0.17a	2.91 ± 0.25a	2.12 ± 0.19a	1.51 ± 0.21b	1.53 ± 0.22b	1.41 ± 0.16b	n.s	*F*_1,156_ = 18.3*	n.s
Litter cover (%)	69.81 ± 5.81b	47.22 ± 4.63b	19.96 ± 5.69b	87.33 ± 1.74a	78.70 ± 3.66a	72.04 ± 3.48a	*F*_2,156_ = 27.4*	*F*_1,156_ = 87.9***	n.s
Ground bryophyte cover (%)	6.56 ± 3.48b	62.00 ± 5.95ab	81.72 ± 5.94a	1.89 ± 0.42bc	3.07 ± 1.17c	2.48 ± 0.64c	*F*_2,156_ = 54.9*	*F*_1,156_ = 240.5***	*F*_2,156_ = 52.6*
Lower grass cover (%)	5.54 ± 1.34b	4.30 ± 1.44b	8.43 ± 3.26b	9.65 ± 1.27a	23.04 ± 3.43a	18.37 ± 2.54a	n.s	*F*_1,156_ = 31.4**	n.s
Lower woody plant cover (%)	1.22 ± 0.53c	4.93 ± 1.51a	3.89 ± 0.88b	1.00 ± 0.63c	8.96 ± 1.36a	3.85 ± 0.70b	*F*_2,156_ = 16.7***	n.s	n.s
Percent of conifer trees	2.96 ± 2.32b	70.20 ± 4.70a	93.57 ± 2.53a	0.37 ± 0.37b	0.19 ± 0.19b	0.56 ± 0.41b	*F*_2,156_ = 194.2***	*F*_1,156_ = 801.0***	*F*_2,156_ = 193.5***

†Variables measured at plot scale and given only for general description of the study sites.

Significance of Permutational ANOVAs is indicated by

* = ≤0.05–0.01

**≤0.01–0.001

***≤0.001. and post hoc significant differences are indicated by different letters.

Permission to access the study sites was given by landowners Daishowa-Marubeni International Ltd and EMEND project leaders, the University of Alberta.

## Data analyses

To assess differences in habitat structure between development stages and cover types, environmental variables were individually compared between the levels of these factors and their interaction using permutational analysis of variance. Significance was assessed by 9999 permutations, shuffling data between stands to account for the nested sampling design. When global tests were significant (P<0.05), pairwise comparisons were carried out between factor levels, correcting P values for multiple testing with the Holm method [29].

Following the approach of [30], total beta diversity was calculated as the total variation in the community data matrix of each arthropod group. We extended this concept to calculate total environmental heterogeneity as the total variation of the environmental variable data matrix. Total variation was calculated as the total sums of squares of the data matrices (i.e. the sum of squared differences of each cell of the matrix from its respective column mean), and this was done separately for mature and regenerating stages. Prior to calculating the sums of squares, environmental variables were scaled to zero mean and unit variance to remove unit effects. At each sampling point, arthropod captures were pooled for the whole study period, then standardised by calculating catch per day to adjust for trap losses, and then multiplied by the median trapping period (83 days) to obtain total captures in the sampling season. Arthropod data were then Hellinger-transformed to reduce double zero effects, decrease the weight of dominant species and render the data appropriate for analyses based on Euclidean distances [29]. The two environmental data matrices (one for mature and one for regenerating stages) were defined by the values of the 16 standardised variables collected at each sampling point. Six arthropod data matrices (one for each arthropod group and development stage combination) were defined by the standardized number of individuals of each species captured at each sampling point.

Sums of squares were calculated on the data matrices and partitioned across cover types, stands and plots using multivariate analysis of variance. Sums of squares corresponding to each scale were divided by their degrees of freedom to obtain variances (or mean squares, as shown in ANOVA tables), which are independent of sample size and thus could be compared within and between development stages. Variances were also calculated for the residual sums of squares, which corresponded to variation within plots (i.e. differences in environmental characteristics or species composition between sampling points).

Variation within each stand was partitioned into the sums of squares for each plot (*within-plots* variation) and the sums of squares of each stand minus the sums of squares for all its plots (*between-plots* variation). Variances *within-* and *between-plots* were then compared between development stages and cover types (and their interaction) using permutational analysis of variance. Significance was assessed by 9999 permutations, and, to account for the nested sampling design in the *within-plots* models, the data were permuted between stands. When global tests were significant (P<0.05), pairwise comparisons were carried out and P values were corrected for multiple testing with the Holm method.

Redundancy analysis (RDA) was used to assess the influence of environmental variables on arthropod beta diversity. Stepwise forward selections were carried out for each arthropod group and development stage. At each step, the environmental variable that explained the highest percentage of variance (r^2^) and was significant (P<0.05, 9999 permutations) was included in the model. Selection stopped when no more significant variables could be added or when the model exceeded the total r^2^ accounted for by the global model [31].

Variation partitioning was used to calculate the percentage of variation at each hierarchical scale (*between-cover types*, *between-stands*, *between-plots* and *within-plots*) that could be explained by the selected environmental variables. Three separate RDA models accounting for the variation *between-cover types*, *between-stands* and *between-plots* were defined using dummy variables. These models were combined with the final environmental models to obtain the percentage of variation explained by environmental variables at each scale. r^2^ values were adjusted in all cases to get unbiased estimators of the variation [32].

Analyses were carried out using package *vegan* [33] in R software [34].

## Results

We captured 6772 spider, 10, 245 staphylinid and 1744 carabid adults from which a total of 143 spider, 87 staphylinid and 24 carabid species were identified. As usual for arthropods, a few very abundant species dominated the assemblages: six species of spider, eight of staphylinid and three of carabid accounted for more than 50% of the total catch, while the 94, 59 and 12 least abundant species together accounted for less than 5% of the total catch (S2, S3 and S4 Tables).

### Characteristics of stands

Total environmental heterogeneity, measured as total variance of the environmental characteristics matrix, was 1.55 times higher in mature (variance = 17.50) than regenerating (variance = 11.31) stands. All mature stands were characterised by tall, thick, widely spaced trees, while regenerating stands had densely packed shorter and thinner stems (Table 1). Soil pH was lowest in mixed and conifer stands in both stages. The soil fermentation layer was deeper in mature than regenerating stands for all cover types whereas the litter layer was only deeper in mature than regenerating conifer stands. In contrast, the percentage cover of litter (comprising leaves, needles and thatch) and lower layer grass was higher in regenerating stands. Finally, conifer and then mixed mature stands had the highest ground layer bryophyte cover, while it did not differ between mature and regenerating stages in deciduous stands. The remaining variables did not differ between cover types, stages and their interaction (S5 Table).

### Contribution of spatial scales to environmental heterogeneity and beta diversity

**Fig 2:**
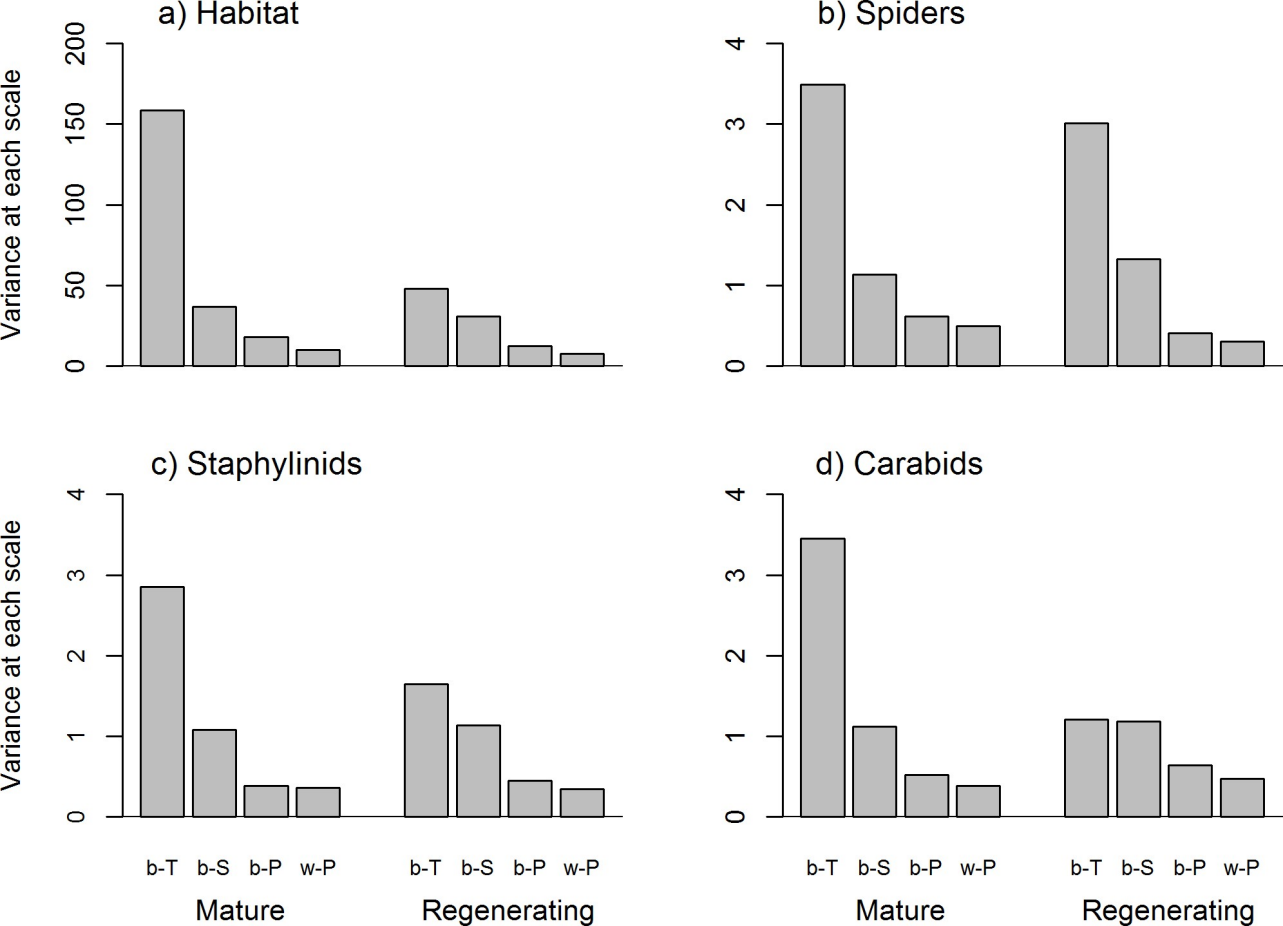
Total variation of a) habitat heterogeneity and b) spider, c) staphylinid and d) carabid beta diversity partitioned across scales: *between-cover types* (b-T), *between-stands* (b-S), *between-plots* (b-P) and *within-plots* (w-P) in mature and regenerating stands.

In general, partition of total environmental heterogeneity and beta diversity for the arthropod groups across scales followed similar patterns: variation *between-cover types* was larger than *between-stands*, particularly in the mature stages, while it decreased gradually *between-* and *within-plots* (Fig 2). *Between-cover* types variation was larger in mature than regenerating stands, particularly for environmental heterogeneity (3.3 times larger), followed by beta diversity of carabids (2.9), staphylinids (1.7) and to a lower extent, spiders (1.2). Differences *between-stands* contributed similarly to environmental heterogeneity and beta diversity in mature and regenerating stages (Fig 2).

### Environmental variables influencing beta diversity in mature and regenerating stands

The environmental variables explained a higher percentage of variation in staphylinid and carabid composition in mature than regenerating stands, however the opposite was true for spiders (Table 2). Different environmental characteristics influenced arthropod beta diversity in mature and regenerating stands; the proportion of conifer trees and soil pH were the most important in mature stands, while ground layer and lower vegetation cover were most relevant in regenerating stands. Environmental models explained a high percentage of the beta diversity due to differences *between-cover types* and *between-stands*, particularly in the mature stage, while they explained little of the beta diversity *between-* and *within-plots* (Table 2).

**Table 2:** Final environmental models and selected environmental variables for each arthropod group and development stage.

		Percentage of variance explained at each scale by the selected environmental variables			
	Total R^2^	*Between-cover types*	*Between-stands*	*Between-plots*	*Within-plots*
**Spiders**					
***Mature*** Percent of conifer trees; Soil pH; Litter soil layer depth (cm); Canopy openness (%); Lower vascular plant cover (%); Tree diversity; Coarse woody debris cover	13.8	77.0	42.1	1.2	2.7
***Regenerating*** Lower vascular plant cover (%); Ground vascular plant cover (%); Lower grass cover; Soil pH; Fine woody debris cover	18.5	69.9	38.3	4.4	3.9
**Staphylinids**					
***Mature*** Percent of conifer trees; Soil pH; Tree diversity; Canopy openness (%); Litter soil layer depth (cm)	16.0	89.6	37.7	0.0	1.7
***Regenerating*** Soil pH; Ground vascular plant cover (%); lower grass cover (%); Canopy openness (%), Tree diversity	9.7	40.9	35.5	8.3	2.8
**Carabids**					
***Mature*** Percent of conifer trees; Soil pH; Fine woody debris cover	18.4	88.6	52.0	25.8	0.0
***Regenerating*** Lower vascular plant cover (%)	1.8	11.3	16.7	4.3	0.0

### Environmental heterogeneity and beta diversity *between-* and *within-plots*

**Fig 3:**
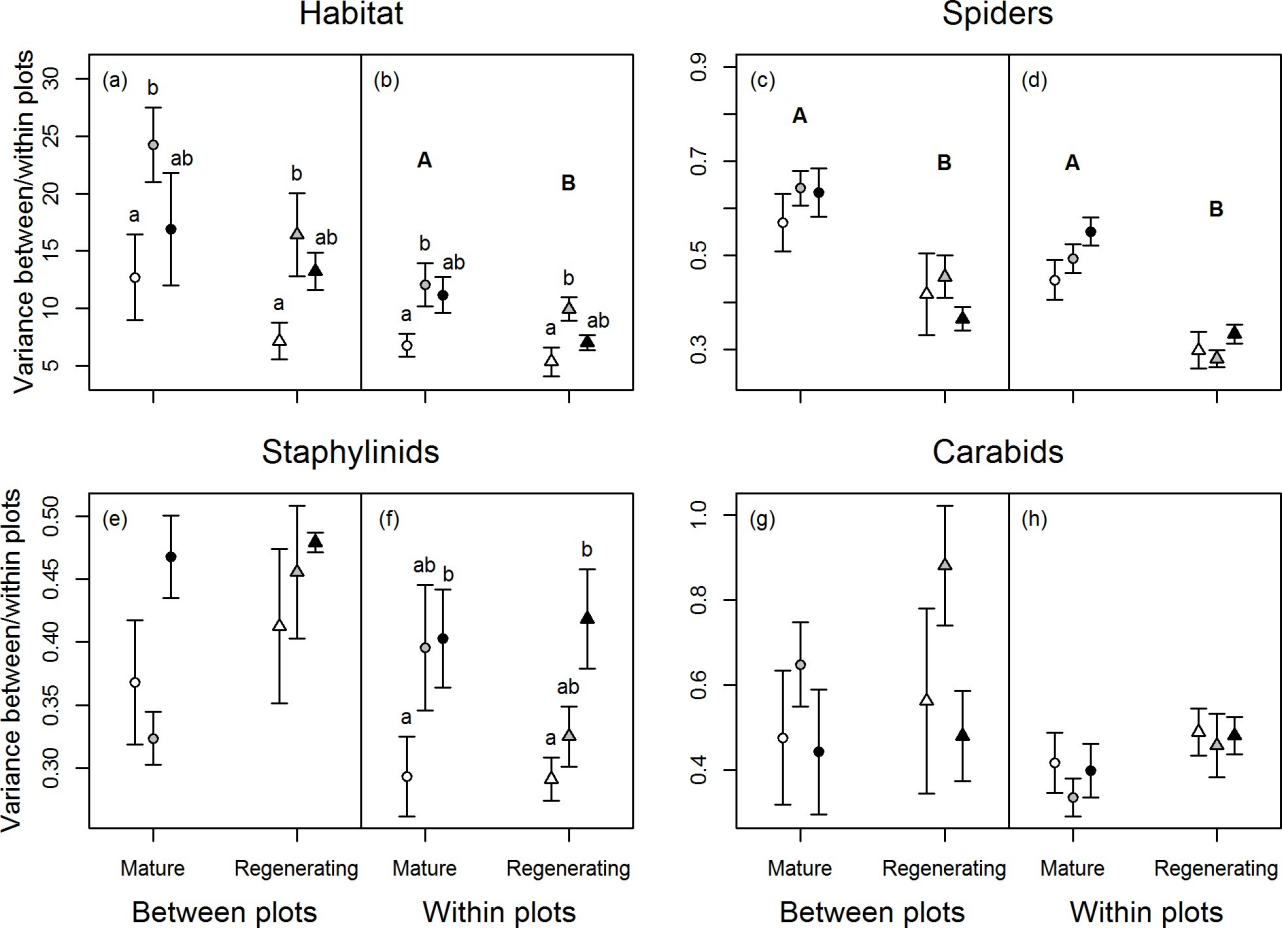
Mean (±SE) sums of squares of habitat characteristics and species composition matrices *between-* and *within-plots* for each cover type x management stage combination. Deciduous stands are indicated by white symbols, mixed stands by grey symbols and coniferous stands by black symbols. Mature stands are indicated by circles and regenerating stands by triangles. Large letters (A,B) indicate significant differences between stages, small letters (a,b) indicate significant differences among cover types as determined by permutational ANOVA.

Environmental heterogeneity and spider beta diversity were higher in mature than regenerating stands at the *within-plots* scale (*F*_1,36_ = 11.16, P = 0.007; *F*_1,36_ = 25.86, P = <0.001 respectively) (Fig 3). Spider beta diversity also followed this trend at the *between-plots* scale (*F*_1,12_ = 20.69, P = 0.002). In contrast, staphylinid and carabid beta diversity did not differ between development stages (Fig 3). Cover type influenced environmental heterogeneity *between-* and *within-plots* (respectively, *F*_2,12_ = 13.98, P = 0.031; *F*_2,36_ = 13.98, P = 0.002) and staphylinid beta diversity *within-plots* (*F*_2,36_ = 4.38, P = 0.032), but spider and carabid beta diversity did not differ significantly across cover types at either spatial scale. Pairwise comparisons showed that environmental heterogeneity was significantly higher in mixed than deciduous stands *within-plots* (P_adj_ = 0.013), and a similar but non-significant trend was found *between-plots* (P_adj_ = 0.066). Staphylinid beta diversity *within-plots* was significantly higher in coniferous than deciduous stands (P_adj_ = 0.021). Interaction terms between development stage and cover type were not significant in any model.

## Discussion

### Scale-dependent influence of forest stage and cover type on environmental heterogeneity

Our results demonstrate that environmental heterogeneity was greater and differently structured in mature than regenerating forests across all spatial scales. At the largest scale (1600-8500m), differences *between-cover types* contributed much more to the total variation in habitat structure in mature than regenerating forest. In mature forests, canopy structure and composition differ among cover types, influencing the amount of light, water, carbon and nutrients available for the understory [35], and in turn, affecting understory plant-species composition and vegetation structure [15]. In contrast, clear-cutting leads to regeneration of aspen at high densities, removing the major structural features among cover types that dominate prior to felling. We suggest that such differences in habitat structure among cover types result in highly heterogeneous mature mixedwood forests, and the corresponding loss of *between-cover type* variability in regenerating forests is a key driver of homogenisation across the post-harvest landscape.

Despite post-harvest homogenisation, we detected a subtle yet notable effect of pre-felling cover type on environmental heterogeneity in the regenerating stands. This indicates that biological legacies linked to pre-felling forest type are retained even 12 years post-harvest, a phenomenon termed ‘ecosystem memory’ by [36]. Our regenerating stands were subject to 98–99% clear-cut and did not retain any significant above ground structures. As such, the ecosystem memory phenomenon may be driven by below ground elements, such as differences in litter or soils linked to the variability in the pre-harvest forest type. Indeed, one such legacy could be soil pH. This factor differed in the same way among cover types in both mature and regenerating stands, and influences ground-dwelling arthropod diversity [37, 38]. Further, pre-harvest forest conditions in the soil or disturbances such as fire can influence factors such as tree regeneration [12, 36], which in turn may influence ground-dwelling arthropods through changes to ground-vegetation and heat and light on the forest floor. This highlights the importance of understanding site history and identifying retained biological legacies when attempting to predict impacts of clear-cutting or other anthropogenic disturbances.

We expected environmental heterogeneity to be greater *between-stands* in mature than in regenerating stands, since the longer growth time gives rise to greater opportunities for diversification in structural attributes. However, this was not the case, and surprisingly environmental heterogeneity was similar in the two forest stages. The higher than expected environmental heterogeneity found among regenerating stands of the same cover type may be explained by differences in geophysical conditions (i.e. topography and lithology), which are particularly influential in the development of young successional stages [12]. Ultimately, this suggests that whilst across landscapes clear-cutting increases environmental homogeneity, this may be less remarkable *between-stands* of the same cover type.

At the *within-plots* scale, (20–40 m), environmental heterogeneity was greater in mature than regenerating stands. A similar, though non-significant trend, was also detected *between-plots* (100-400m). In mature stands, canopy and understory vegetation are patchily distributed at small scales, the understory being particularly influenced by individual tree canopies [39]. On the contrary, patchiness in understory resources may be low in regenerating stands, in part due to the uniform cover of young aspen stems, resulting in low environmental heterogeneity.

In mature stands, environmental heterogeneity differed at small scales (20-40m and 100–400 m) among cover types: it was greater in mixed than deciduous stands, and intermediate in conifers. In mixed stands, areas associated with deciduous and coniferous trees support different understory communities [39], which may enhance environmental heterogeneity at within stands. Interestingly, in regenerating stands environmental heterogeneity also differed among pre-harvest cover types at this small scale, reflecting the same pattern of difference as mature stands, albeit to a lower magnitude. Again, this may indicate the importance of ecosystem memory in shaping environmental heterogeneity in post-harvest systems.

### Scale-dependent influences of forest stage and cover type on arthropod beta diversity

Beta diversity reflected environmental heterogeneity patterns *between-cover types* and *between-stands*. Like environmental heterogeneity, total beta diversity was higher in mature than regenerating stands largely due to the notable variation among cover types. Environmental conditions in mature stands differed widely among cover types, supporting different arthropod assemblages. Most of this variation was reflected by the environmental variables selected by the redundancy analyses, with the proportion of conifer stems being particularly important. Conifer canopies affect light and water availability, litter amount and composition, and soil nutrients differently than deciduous canopies [38], strongly influencing understory plant communities [15, 39] and ground dwelling arthropod assemblages [17, 21]. Soil pH and litter depth also influenced species composition in mature stands. Soil pH can affect habitat selection of ground-dwelling arthropods either directly, as occurs for some carabid species [37] or indirectly by influencing habitat selection of their prey items such as springtails, mites and woodlice [40]. Further, soil pH may affect plant species composition, and this in turn influences microclimatic conditions. Differences in litter depth affect spider abundance and richness [41, 42], and carabid distribution patterns [43] through abiotic (temperature, humidity) and biotic factors (e.g., improved food supply).

As expected, the influence of forest cover type on arthropod beta diversity was lower in regenerating than mature stands, however, the effect of pre-harvest type among regenerating stands was more pronounced than that observed for environmental heterogeneity. This was particularly notable for spiders where the variation attributed to cover type was similar in mature and regenerating stands, suggesting a strong ecosystem memory effect for this group. Spider assemblages in regenerating stands were strongly influenced by cover of lower vascular plants; this was also true for staphylinid and carabid beetles, albeit to a lesser extent. Lower vegetation layers are important determinants of ground-dwelling arthropod diversity influencing food supply (e.g., prey or seed availability) or structures useful to hunters such as provision of web attachment points [22, 44, 45]. In regenerating stands, lower vegetation cover may be limited by the density of aspen suckers, which strongly compete with them for resources, and is in turn influenced by pre-harvest forest cover types [46]. This again highlights the important role of site history when predicting faunal recovery following disturbance [36].

At smaller scales, *between- and within-plots* (100–400 m and 20–40 m), spider beta diversity was higher in mature than regenerating stands, reflecting overall trends in environmental heterogeneity. In regenerating stands, low environmental heterogeneity at these scales may have homogenised spider assemblages, since their diversity depends to some extent on the complexity of litter and vegetation structure [41, 44]. This was not the case for staphylinid and carabid beetles however, as they showed no difference in beta diversity between mature and regenerating stages, indicating that the environmental homogenisation we detected following clear-cut is a less important determinant of their beta diversity at these scales.

Beta diversity did not reflect trends in environmental heterogeneity between cover types. The latter was greatest in mixed stands at *between- and within-plots* scales, whereas beta diversity was similar for carabids and spiders across the cover types, and for staphylinids it was highest in conifers. Staphylinid beetle responses to important ‘keystone’ features [11] related to soil, litter or dead wood may explain such differences. For instance, the conifer stands had the deepest litter and many staphylinid species are associated with different rotting materials like thick litter layers, dead fungi and corpses where they feed on saprophagous insects, micro fungi or directly on rotting material [47]. [15] found that plant beta diversity also increased along the successional gradient from deciduous to conifer in regenerating as well as mature stands in the same study area, perhaps reflecting retention of biological legacies (e.g. soil parameters) after clear-cutting. Further, we found that proportion of conifer trees (and factors related to it, e.g., soil pH), were selected as a significant determinant of beta diversity in the models for all arthropod groups in mature stands. Taken together, these results underscore the important influence of conifers on biodiversity in boreal mixedwoods, both before and after clear-cutting.

Finally, it is notable that carabid beta diversity did not reflect trends in environmental heterogeneity or differ among cover types or developmental stages at *between-* and *within-plots* scales, even though carabids assemblages are known to be highly heterogenous at small scales in the boreal forest [17, 20]. This may be attributed to low numbers of carabid species sampled (relative to other the other taxa), limiting our ability to detect differences in beta diversity among forest types. Or, it may also be that the movement of individuals in search of favourable resources [20] is a key driver of beta diversity at these scales, irrespective of the forest type. This suggests better knowledge of species mobility and resource requirements, which is generally lacking for arthropods, would aid to the understanding of drivers of beta diversity at the smallest scales.

## Conclusions

Overall, arthropod beta diversity reflected the patterns of environmental heterogeneity at the larger scales (*between cover types* and *between stands*) but there was little relationship at smaller scales (*between- and within-plots*). Beta diversity at smaller scales may be influenced by movement of individuals, dispersal pressures, neutral effects, variability in key stone structures or sampling artefacts.

Our results show that clear-cutting alters environmental heterogeneity across the largest spatial scale, leading to more homogenous forest landscapes. However, whilst this was reflected in spiders beta diversity, staphylinid and carabid beetle assemblages were not homogenised after clear-cutting. Moreover, in regenerating stands pre-harvest cover type influenced environmental heterogeneity and arthropod beta diversity, suggesting that retained biological legacies are likely drivers of post-harvest beta diversity patterns at large scales in all three arthropod groups, but also at smaller scales for staphylinid beetles.

Interestingly, beta diversity of beetles did not strongly reflect patterns of environmental heterogeneity across spatial scales, as was shown for spiders. We defined environmental heterogeneity through a range of environmental factors that are easy to measure and often used to describe forest condition (e.g., DBH, stem density, canopy openness [9] or known to be important for ground-dwelling arthropod diversity (e.g., vegetation cover, litter depth, soil pH). For spiders, which are primarily influenced by changes in habitat structure [45], these variables were adequate predictors of beta diversity patterns, but for beetles, other resources may be more strongly associated with beta diversity and ways to determine and measure these should be explored. However, in the absence of effective tools to manage small-scale variation, promoting forests with stands of different cover types, including later successional stages, is a sound conservation strategy.

Although clear-cutting is a more uniform than most natural disturbances, it seems that biological legacies contribute to heterogeneity of species and the forest environment even 12 years post-harvest. Although some work has addressed harvest emulations of stand-replacing natural disturbances [48, 49], better understanding of the role of biological legacies in shaping post-harvest communities will contribute to more effective forest management, particularly by supporting selection of appropriate target stands for development to late successional stages.

## Supporting information

S1 Table Reference list of the keys used for arthropod identification and the species catalogues adopted for nomenclature. (DOCX)

S2 Table Spiders collected at deciduous-dominated (DD), mixed (MX) and coniferous-dominated (CD) mature and regenerating forests. (DOCX)

S3 Table Staphylinids collected at deciduous-dominated (DD), mixed (MX) and coniferous-dominated (CD) mature and regenerating forests. (DOCX)

S4 Table Carabids collected at deciduous-dominated (DD), mixed (MX) and coniferous-dominated (CD) mature and regenerating forests. (DOCX)

S5 Table Environmental characteristics (mean ± SE) that did not differ significantly by cover type (deciduous-dominated (DD), mixed (MX) and conifer-dominated (CD) stands), stage (mature and regenerating forests) and their interaction. (DOCX)
