# Supplementary material for: Natural succession and clearcutting as drivers of environmental heterogeneity and beta diversity in North American boreal forests
Source: PLoS One. 2018 Nov 2;13(11):e0206931. doi: 10.1371/journal.pone.0206931 (PMC6214561; doi:10.1371/journal.pone.0206931)
Supplement: S1 Table — (DOCX) [file pone.0206931.s001.docx]

**S1 Table Reference list of the keys used for arthropod identification and the species catalogues adopted for nomenclature.**

**Spiders**

Dondale, C. D. & J. H. Redner. 1978. *The insects and arachnids of Canada. Part 5. The crab spiders of Canada and Alaska: Philodromidae and Thomisidae*. Agriculture Canada, Ottawa, 255 pp.

Dondale, C. D. & J. H. Redner. 1982. *The insects and arachnids of Canada. Part 9. The sac spiders of Canada and Alaska: Clubionidae and Anyphaenidae*. Agriculture Canada, Ottawa, 194 pp.

Dondale, C. D. & J. H. Redner. 1990. *The insects and arachnids of Canada. Part 17. The wolf spiders, nurseryweb spiders and lynx spiders of Canada and Alaska: Lycosidae, Pisauridae and Oxyopidae*. Agriculture Canada, Ottawa, 383 pp.

Dondale, C. D., J. H. Redner, P. Paquin*, et al.* 2003. *The insect and arachnids of Canada. Part 23. The orb-weaving spiders of Canada and Alaska: Uloboridae, Tetragnathidae, Araneidae and Theridiosomatidae*. Agriculture Canada, Ottawa, 371 pp.

Paquin, P. & N. Dupérre. 2003. *Guide d'indentification des araignées (Araneae) du Québec*. Fabreries, Supplement 11, 251 pp.

Pinzón, J. 2011. Composition and structure of spider assemblages in layers of the mixedwood boreal forest after variable retention harvest. Appendix 1: An illustrated guide to the sheet-weaving spiders (Family Linyphiidae) of Alberta. PhD Thesis. University of Alberta.

Platnick, N. I. 2011. The World Spider Catalog, Version 11.5. American Museum of Natural History, Online at: http://research.amnh.org/entomology/spiders/catalog/.

Platnick, N. I. & C. D. Dondale. 1992. *The insects and arachnids of Canada. Part 19. The ground spiders of Canada and Alaska: Gnaphosidae*. Agriculture Canada, Ottawa, 297 pp.

**Staphylinids**

Assing, V., Wunderle, P. 1995. A revision of the species of the subfamily Habrocerinae (Coleoptera: Staphylinidae) of the world. Revue suisse de Zoologie 102: 307-359.

Brunke, A., Newton, A., Klimaszewski, J., Majka, C., Marshall, S. 2011. Staphylinidae of Eastern Canada and Adjacent United States. Key to Subfamilies; Staphylinidae: Tribes and Subtribes, and Species of Staphylinina. Canadian Journal of Arthropod Identification 12: 1-110.

Campbell, J.M. 1968. A revision of the New World Micropeplinae (Coleoptera: Staphylinidae) with a rearrangement of the World species. Can. Entomol. 100: 225-267.

Campbell, J.M. 1973. A revision of the genus *Tachinus* (Coleoptera: Staphylinidae) of North and Central America. Memoirs of the Entomological Society of Canada. 90: 1-137.

Campbell, J.M. 1975. New species and records of *Tachinus* (Coleoptera: Staphylinidae) from North America. Can. Entomol. 107: 87-94.

Campbell, J.M. 1978. New species of *Oxyporus* (Coleoptera: Staphylinidae) from North America. Can. Entomol. 110: 805-813.

Campbell, J.M. 1979. A revision of the genus *Tachyporus* Gravenhorst (Coleoptera: Staphylinidae) of North and Central America. Memoirs of the Entomological Society of Canada, 109: 1-95.

Campbell, J.M. 1982. A revision of the genus *Lordithon* Thomson of North and Central America (Coleoptera: Staphylinidae). Memoirs of the Entomological Society of Canada 114 (suppl. S119): 5-116.

Campbell, J.M. 1983. A new species of *Pycnoglypta* Thomson (Coleoptera: Staphylinidae) from eastern Canada. Can. Entomol. 115: 361-370.

Campbell, J.M. 1983. A revision of the North American Omaliinae (Coleoptera: Staphylinidae). The genus *Acidota* Stephens. Can. Entomol. 114: 1003-1029.

Campbell, J.M. 1983. A revision of the North American Omaliinae (Coleoptera: Staphylinidae). The genus *Olophrum* Erichson. Can. Entomol. 115: 577-622.

Campbell, J.M. 1984. A revision of the North American Omaliinae (Coleoptera: Staphylinidae). The genera *Arpedium* Erichson and *Eucnecosum* Reitter. Can. Entomol. 116: 487-527.

Campbell, J.M. 1991. A revision of the genera *Mycetoporus* Mannerheim and *Ischnosoma* Stephens (Coleoptera: Staphylinidae: Tachyporinae) of North and Central America. Memoirs of the Entomological Society of Canada 123 (Suppl. S156): 3-169.

Campbell, J.M. 1993. A revision of the genera *Bryoporus* Kraatz and *Bryophacis* Reitter and two new related genera from America North of Mexico (Coleoptera: Staphylinidae: Tachyporinae). Memoirs of the Entomological Society of Canada 166: 85 pp.

Campbell, J.M. 1993. A review of the species of *Nitidotachinus* new genus (Coleoptera: Staphylinidae: Tachyporinae). Can. Entomol. 125: 521-548.

Casey, T.L. 1884. A revision of the Stenini of America North of Mexico, order Coleoptera. Collins Printing House, Philadelphia, PA. 206 pp.

Casey, T.L. 1905. A revision of the American Paederini. Trasactions of the Academy of Science of St. Louis. 15: 17-434.

Cuccodoro, G.; Löbl, I. 1996. Revision of the rove-beetles of the genus *Megarthrus* of America north of Mexico. Mitt. Münch. Ent. Ges. 86: 145-188.

Hatch, M.H. 1957. The Beetles of the Pacific Northwest, Part II: Staphyliniformia. University of Washington Publications in Biology, Vol. 16, Seattle, WA. 384 pp.

Herman, L.H. 2001. Catalog of the Staphylinidae (Insecta: Coleoptera). 1758 to the end of the second millennium. Parts I-VII. Bulletin of the American Museum of Natural History 265: 1-4218.

Moore, I. 1975. The distribution of *Siagonium* (Coleoptera: Staphylinidae) in North America. J. Kansas Entomol. Soc. 48: 96-100.

Newton, A. F., M. K. Thayer, J. S. Ashe, and D. S. Chandler. 2001. Staphylinidae. pp. 268-418 *in* Arnett, R.H., Jr., and M. C. Thomas. (eds.). American Beetles. Volume 1. Archostemata, Myxophaga, Adephaga, Polyphaga: Staphyliniformia. CRC Press LLC, Boca Raton, FL. xvi + 443 pp.

Outerelo, R., Gamarra, P., de la Rosa, J.J., Marín Armijos, D. 2010. *Phloeostiba lapponica* (Zetterstedt, 1838) especie boreal, nueva para la fauna de la Península Ibérica (Coleoptera, Staphylinidae, Omaliinae). Boletín de la Sociedad Entomológica Aragonesa (S.E.A.) 46: 553-555.

Smetana, A. 1985. Systematic position and review of *Deinopteroloma* Jansson, 1946, with descriptions of four new species (Coleoptera, Silphidae and Staphylinidae (Omaliinae)). Systematic Entomology 10: 471-499.

Smetana, A. 1995. Rove beetles of the subtribe Philonthina of America north of Mexico (Coleoptera: Staphylinidae). Classification, phylogeny and taxonomic revision. Memoirs of Entomology, International, 3: x + 946 pp.

Steel, W.O. 1957. Notes on the Omaliinae (Col. Staphylinidae). The genus *Acrolocha* Thomson. Entomol. Monthly. Mag. 93: 157-164.

**Carabids**

Bousquet, Y., Larochelle, A. 1993. Catalogue of the Geadephaga (Coleoptera: Trachypachidae, Rhysodidae, Carabidae including Cicindelini) of America north of Mexico. Memoirs of the Entomological Society of Canada 167. 397 pp.

Lindroth, C.H., 1961, 1963, 1966, 1968, 1969, 1969. The ground-beetles (Carabidae, excl. Cicindelinae) of Canada and Alaska, Parts 1-6. Opusc. Entomol. Suppl. 20, 1-200; 24, 201-408; 29, 409-648; 33, 649-944; 34, 945-1192; 35, I-XLVIII.
