# Supplementary material for: Natural succession and clearcutting as drivers of environmental heterogeneity and beta diversity in North American boreal forests
Source: PLoS One. 2018 Nov 2;13(11):e0206931. doi: 10.1371/journal.pone.0206931 (PMC6214561; doi:10.1371/journal.pone.0206931)
Supplement: S2 Table — (DOCX) [file pone.0206931.s002.docx]

**S2. Table Spiders collected at deciduous-dominated (DD), mixed (MX) and coniferous-dominated (CD) mature and regenerating forests.**

|  | **Mature** | | | |  | **Regenerating** | | | **Total** |
| --- | --- | --- | --- | --- | --- | --- | --- | --- | --- |
|  | **DD** | **MX** | **CD** | |  | **DD** | **MX** | **CD** |  |
| *Agelenopsis utahana* | 10 | 1 | 1 | |  | 5 | 6 | 3 | 26 |
| *Agroeca ornata* | 9 | 14 | 14 | |  | 2 | 5 | 1 | 45 |
| *Agyneta allosubtilis* | 3 | 4 | 3 | |  | 1 | 1 | 7 | 19 |
| *Agyneta olivacea* | 15 | 7 | 3 | |  | 58 | 99 | 94 | 276 |
| *Allomengea dentisetis* | 140 | 49 | 28 | |  | 139 | 70 | 34 | 460 |
| *Alopecosa aculeata* | 1 | 1 | 1 | |  | 5 | 63 | 107 | 178 |
| *Amaurobius borealis* | 6 | 3 | 2 | |  | 5 | 6 | 1 | 23 |
| *Aphileta misera* | 0 | 0 | 0 | |  | 1 | 0 | 0 | 1 |
| *Araneus saevus* | 0 | 0 | 0 | |  | 0 | 0 | 1 | 1 |
| *Arctobius agelenoides* | 0 | 0 | 2 | |  | 0 | 0 | 0 | 2 |
| *Arctosa raptor* | 0 | 0 | 1 | |  | 0 | 0 | 1 | 2 |
| *Baryphyma gowerense* | 0 | 0 | 0 | |  | 1 | 0 | 0 | 1 |
| *Bathyphantes brevipes* | 0 | 0 | 2 | |  | 2 | 0 | 2 | 6 |
| *Bathyphantes brevis* | 0 | 0 | 2 | |  | 0 | 0 | 1 | 3 |
| *Bathyphantes pallidus* | 41 | 4 | 6 | |  | 60 | 106 | 108 | 325 |
| *Callobius nomeus* | 0 | 3 | 0 | |  | 0 | 0 | 0 | 3 |
| *Ceraticelus fissiceps* | 11 | 1 | 1 | |  | 21 | 14 | 9 | 57 |
| *Ceratinella brunnea* | 0 | 0 | 0 | |  | 2 | 0 | 3 | 5 |
| *Clubiona canadensis* | 10 | 5 | 1 | |  | 4 | 0 | 1 | 21 |
| *Clubiona directa* | 0 | 1 | 0 | |  | 0 | 0 | 0 | 1 |
| *Clubiona euopla* | 0 | 1 | 0 | |  | 0 | 0 | 0 | 1 |
| *Clubiona furcata* | 0 | 0 | 0 | |  | 1 | 3 | 0 | 4 |
| *Clubiona kulczynskii* | 2 | 0 | 3 | |  | 5 | 6 | 1 | 17 |
| *Clubiona opeongo* | 0 | 0 | 0 | |  | 0 | 3 | 0 | 3 |
| *Cnephalocotes obscurus* | 0 | 0 | 1 | |  | 0 | 0 | 1 | 2 |
| *Crustulina stricta* | 0 | 0 | 0 | |  | 0 | 1 | 0 | 1 |
| *Cryphoeca montana* | 0 | 1 | 0 | |  | 0 | 0 | 0 | 1 |
| *Cybaeopsis euopla* | 37 | 3 | 12 | |  | 81 | 27 | 8 | 168 |
| *Diplocentria bidentata* | 57 | 98 | 79 | |  | 126 | 63 | 60 | 483 |
| *Diplocentria perplexa* | 0 | 2 | 2 | |  | 0 | 3 | 0 | 7 |
| *Diplocentria rectangulata* | 1 | 6 | 21 | |  | 0 | 2 | 0 | 30 |
| *Dismodicus bifrons* | 0 | 0 | 0 | |  | 1 | 0 | 0 | 1 |
| *Dismodicus decemaculatus* | 0 | 0 | 2 | |  | 3 | 0 | 1 | 6 |
| *Drassodes neglectus* | 0 | 0 | 0 | |  | 0 | 2 | 1 | 3 |
| *Estrandia grandaeva* | 1 | 0 | 0 | |  | 0 | 0 | 0 | 1 |
| *Evarcha hoyi* | 0 | 0 | 0 | |  | 0 | 1 | 0 | 1 |
| *Gnaphosa borea* | 2 | 3 | 2 | |  | 6 | 77 | 98 | 188 |
| *Gnaphosa brumalis* | 0 | 0 | 0 | |  | 0 | 1 | 0 | 1 |
| *Gnaphosa hyperborea* | 0 | 0 | 1 | |  | 0 | 4 | 0 | 5 |
| *Gnaphosa microps* | 3 | 6 | 8 | |  | 7 | 62 | 102 | 188 |
| *Gnaphosa muscorum* | 0 | 0 | 0 | |  | 1 | 2 | 1 | 4 |
| *Gnaphosa parvula* | 5 | 3 | 1 | |  | 7 | 27 | 22 | 65 |
| *Gonatium crassipalpum* | 0 | 0 | 0 | |  | 0 | 2 | 4 | 6 |
| *Grammonota angusta* | 0 | 0 | 1 | |  | 0 | 0 | 1 | 2 |
| *Grammonota gigas* | 0 | 0 | 0 | |  | 9 | 9 | 5 | 23 |
| **Supplementary Table S2**. *Continued* |  |  |  | |  |  |  |  |  |
|  | **Mature** | | |  | **Regenerating** | | | **Total** |  |
|  | **DD** | **MX** | **CD** |  | **DD** | **MX** | **CD** |  |  |
| *Haplodrassus hiemalis* | 0 | 0 | 0 |  | 1 | 6 | 5 | 12 |  |
| *Haplodrassus signifer* | 0 | 0 | 0 |  | 0 | 1 | 0 | 1 |  |
| *Helophora insignis* | 2 | 0 | 0 |  | 1 | 0 | 1 | 4 |  |
| *Hilaira canaliculata* | 0 | 0 | 0 |  | 1 | 0 | 0 | 1 |  |
| *Hilaira herniosa* | 0 | 3 | 2 |  | 0 | 1 | 0 | 6 |  |
| *Hybauchenidium gibbosum* | 40 | 3 | 2 |  | 347 | 58 | 67 | 517 |  |
| *Hyposinga rubens* | 0 | 0 | 0 |  | 0 | 0 | 1 | 1 |  |
| *Hypselistes florens* | 0 | 0 | 1 |  | 1 | 3 | 3 | 8 |  |
| *Incestophantes complicatus* | 1 | 13 | 18 |  | 12 | 9 | 6 | 59 |  |
| *Incestophantes duplicatus* | 1 | 5 | 1 |  | 0 | 0 | 0 | 7 |  |
| *Kaestneria pullata* | 0 | 0 | 0 |  | 4 | 0 | 0 | 4 |  |
| *Lepthyphantes alpinus* | 19 | 34 | 45 |  | 6 | 4 | 0 | 108 |  |
| *Lepthyphantes directa* | 0 | 0 | 3 |  | 0 | 0 | 0 | 3 |  |
| *Macrargus multisemius* | 0 | 0 | 0 |  | 0 | 2 | 2 | 4 |  |
| *Maro amplus* | 1 | 1 | 0 |  | 0 | 1 | 1 | 4 |  |
| *Maso sundevalli* | 0 | 1 | 1 |  | 3 | 2 | 1 | 8 |  |
| *Meioneta simplex* | 0 | 0 | 0 |  | 0 | 6 | 9 | 15 |  |
| *Micaria aenea* | 0 | 0 | 0 |  | 1 | 0 | 8 | 9 |  |
| *Micaria pulicaria* | 0 | 0 | 0 |  | 0 | 2 | 4 | 6 |  |
| *Microlinyphia pusilla* | 0 | 0 | 0 |  | 0 | 0 | 1 | 1 |  |
| *Microneta viaria* | 13 | 1 | 0 |  | 3 | 5 | 0 | 22 |  |
| *Neon nelli* | 4 | 0 | 0 |  | 0 | 0 | 0 | 4 |  |
| *Oedothorax gibbosus* | 4 | 0 | 0 |  | 0 | 0 | 0 | 4 |  |
| *Oedothorax trilobatus* | 0 | 0 | 0 |  | 1 | 0 | 0 | 1 |  |
| *Oreonetides rectangulatus* | 0 | 0 | 0 |  | 2 | 0 | 0 | 2 |  |
| *Oreonetides vaginatus* | 12 | 8 | 7 |  | 6 | 6 | 1 | 40 |  |
| *Orodrassus canadensis* | 0 | 1 | 1 |  | 0 | 0 | 0 | 2 |  |
| *Oryphantes aliquantulus* | 0 | 0 | 2 |  | 0 | 1 | 5 | 8 |  |
| *Oryphantes rectangulatus* | 0 | 0 | 0 |  | 1 | 0 | 0 | 1 |  |
| *Ozyptila sincera canadensis* | 1 | 1 | 0 |  | 6 | 6 | 4 | 18 |  |
| *Pardosa exigua* | 0 | 0 | 0 |  | 0 | 0 | 1 | 1 |  |
| *Pardosa fuscula* | 0 | 0 | 0 |  | 1 | 0 | 0 | 1 |  |
| *Pardosa hyperborea* | 0 | 0 | 0 |  | 2 | 52 | 61 | 115 |  |
| *Pardosa mackenziana* | 41 | 11 | 5 |  | 32 | 48 | 148 | 285 |  |
| *Pardosa moesta* | 12 | 10 | 5 |  | 408 | 442 | 464 | 1341 |  |
| *Pardosa uintana* | 0 | 0 | 7 |  | 0 | 0 | 2 | 9 |  |
| *Pardosa xerampelina* | 0 | 0 | 1 |  | 3 | 44 | 51 | 99 |  |
| *Pelecopsis mengei* | 0 | 1 | 6 |  | 0 | 4 | 13 | 24 |  |
| *Pelecopsis sculpta* | 0 | 15 | 2 |  | 0 | 2 | 0 | 19 |  |
| *Pelegrina flavipes* | 0 | 0 | 2 |  | 0 | 0 | 0 | 2 |  |
| *Pelegrina montana* | 0 | 0 | 0 |  | 0 | 0 | 1 | 1 |  |
| *Philodromus pernius* | 0 | 1 | 0 |  | 0 | 0 | 0 | 1 |  |
| *Pirata byzantae* | 0 | 0 | 0 |  | 1 | 1 | 1 | 3 |  |
| *Pityophantes subarcticus* | 1 | 2 | 0 |  | 1 | 0 | 0 | 4 |  |
| *Pocadicnemis occidentalis* | 0 | 5 | 4 |  | 3 | 40 | 57 | 109 |  |
| *Porrhomma terrestre* | 5 | 0 | 0 |  | 6 | 0 | 1 | 12 |  |
| *Robertus fuscus* | 1 | 5 | 0 |  | 2 | 1 | 0 | 9 |  |
| *Rugathodes aurantius* | 24 | 25 | 19 |  | 37 | 0 | 1 | 106 |  |
| **Supplementary Table S2**. *Continued.* | | | | | | | | |  |
|  | Mature | | |  | Regenerating | | | Total |  |
|  | DD | MX | CD |  | DD | MX | CD |  |  |
| *Rugathodes sexpunctatus* | 0 | 2 | 5 |  | 5 | 0 | 0 | 12 |  |
| *Sciastes dubius* | 0 | 1 | 0 |  | 0 | 0 | 0 | 1 |  |
| *Sciastes truncatus* | 7 | 6 | 7 |  | 13 | 9 | 3 | 45 |  |
| *Scironis tarsalis* | 0 | 1 | 0 |  | 8 | 2 | 4 | 15 |  |
| *Scotinotylus sacer* | 0 | 4 | 2 |  | 0 | 0 | 0 | 6 |  |
| *Semiljicola obtusus* | 0 | 0 | 0 |  | 0 | 1 | 0 | 1 |  |
| *Sisicus apertus* | 1 | 3 | 0 |  | 1 | 0 | 2 | 7 |  |
| *Sisis rotundus* | 0 | 0 | 1 |  | 0 | 0 | 0 | 1 |  |
| *Sissicottus montanus* | 7 | 23 | 11 |  | 2 | 1 | 3 | 47 |  |
| *Styloctetor stavivus* | 0 | 0 | 0 |  | 0 | 0 | 3 | 3 |  |
| *Tapinocyba cameroni* | 0 | 0 | 4 |  | 1 | 1 | 0 | 6 |  |
| *Tapinocyba prima* | 0 | 0 | 0 |  | 0 | 0 | 1 | 1 |  |
| *Tapinocyba simplex* | 0 | 0 | 0 |  | 0 | 0 | 1 | 1 |  |
| *Tetragnatha versicolor* | 0 | 1 | 0 |  | 0 | 0 | 0 | 1 |  |
| *Thanatus formicinus* | 0 | 0 | 0 |  | 0 | 0 | 1 | 1 |  |
| *Theridiidae impressum* | 0 | 0 | 0 |  | 0 | 1 | 0 | 1 |  |
| *Theridion ohlerti* | 1 | 0 | 0 |  | 0 | 0 | 0 | 1 |  |
| *Tibellus maritimus* | 0 | 0 | 0 |  | 1 | 0 | 0 | 1 |  |
| *Tibellus oblongus* | 0 | 0 | 0 |  | 0 | 1 | 0 | 1 |  |
| *Titanoeca nivalis* | 0 | 0 | 0 |  | 0 | 1 | 0 | 1 |  |
| *Trochosa terricola* | 1 | 0 | 0 |  | 2 | 6 | 6 | 15 |  |
| *Tunagyna debilis* | 0 | 0 | 0 |  | 2 | 1 | 6 | 9 |  |
| *Vermontia thoracia* | 0 | 0 | 5 |  | 1 | 0 | 0 | 6 |  |
| *Walckenaeria aimakensis* | 0 | 0 | 0 |  | 1 | 0 | 0 | 1 |  |
| *Walckenaeria arctica* | 0 | 0 | 1 |  | 0 | 4 | 0 | 5 |  |
| *Walckenaeria atrotibialis* | 17 | 9 | 12 |  | 32 | 18 | 23 | 111 |  |
| *Walckenaeria auranticeps* | 0 | 0 | 0 |  | 2 | 2 | 0 | 4 |  |
| *Walckenaeria castanea* | 23 | 11 | 10 |  | 26 | 43 | 21 | 134 |  |
| *Walckenaeria communis* | 1 | 43 | 29 |  | 0 | 5 | 5 | 83 |  |
| *Walckenaeria cuspidata brevicula* | 0 | 0 | 4 |  | 1 | 0 | 4 | 9 |  |
| *Walckenaeria directa* | 28 | 5 | 17 |  | 22 | 29 | 20 | 121 |  |
| *Walckenaeria exigua* | 0 | 0 | 0 |  | 2 | 5 | 9 | 16 |  |
| *Walckenaeria fallax* | 0 | 1 | 0 |  | 0 | 0 | 0 | 1 |  |
| *Walckenaeria karpinskii* | 0 | 25 | 5 |  | 0 | 4 | 1 | 35 |  |
| *Walckenaeria kochi* | 0 | 1 | 0 |  | 0 | 0 | 0 | 1 |  |
| *Walckenaeria lepida* | 0 | 0 | 0 |  | 1 | 0 | 0 | 1 |  |
| *Walckenaeria minuta* | 0 | 0 | 0 |  | 0 | 3 | 0 | 3 |  |
| *Walckenaeria palllida* | 0 | 0 | 0 |  | 1 | 0 | 0 | 1 |  |
| *Walckenaeria spiralis* | 0 | 0 | 0 |  | 0 | 3 | 0 | 3 |  |
| *Walckenaeria tricornis* | 0 | 1 | 1 |  | 0 | 1 | 3 | 6 |  |
| *Walckenaeria vigilax* | 0 | 0 | 2 |  | 0 | 0 | 0 | 2 |  |
| *Walckenaria directa* | 0 | 0 | 0 |  | 0 | 1 | 0 | 1 |  |
| *Xysticus canadensis* | 2 | 34 | 36 |  | 0 | 0 | 0 | 72 |  |
| *Xysticus ellipticus* | 0 | 0 | 0 |  | 3 | 4 | 4 | 11 |  |
| *Xysticus emertoni* | 0 | 0 | 0 |  | 10 | 30 | 8 | 48 |  |
| *Xysticus luctuosus* | 1 | 0 | 1 |  | 2 | 1 | 3 | 8 |  |
| *Xysticus obscurus* | 8 | 7 | 9 |  | 0 | 0 | 1 | 25 |  |
|  |  |  |  |  |  |  |  |  |  |
|  |  |  |  |  |  |  |  |  |  |
| **Supplementary Table S2**. *Continued.* | | | | | | | | |  |
|  | Mature | | |  | Regenerating | | | Total |  |
|  | DD | MX | CD |  | DD | MX | CD |  |  |
| *Zelotes fratris* | 0 | 0 | 0 |  | 0 | 5 | 9 | 14 |  |
| *Zelotes puritanus* | 0 | 0 | 0 |  | 0 | 0 | 1 | 1 |  |
| *Zornella armata* | 23 | 51 | 8 |  | 24 | 73 | 6 | 185 |  |
| Number of individuals | 656 | 591 | 504 |  | 1608 | 1667 | 1746 | 6772 |  |
| Number of species | 48 | 60 | 66 |  | 75 | 79 | 81 | 143 |  |
