# Supplementary material for: Natural succession and clearcutting as drivers of environmental heterogeneity and beta diversity in North American boreal forests
Source: PLoS One. 2018 Nov 2;13(11):e0206931. doi: 10.1371/journal.pone.0206931 (PMC6214561; doi:10.1371/journal.pone.0206931)
Supplement: S3 Table — (DOCX) [file pone.0206931.s003.docx]

**S3. Table Staphylinids collected at deciduous-dominated (DD), mixed (MX) and coniferous-dominated (CD) mature and regenerating forests.**

|  | **Mature** | | |  | **Regenerating** | | | | **Total** |
| --- | --- | --- | --- | --- | --- | --- | --- | --- | --- |
|  | **DD** | **MX** | **CD** |  | **DD** | **MX** | **CD** | |  |
| *Acidota crenata* | 0 | 1 | 1 |  | 0 | 1 | 2 | | 5 |
| *Acidota quadrata* | 98 | 49 | 36 |  | 415 | 227 | 128 | | 953 |
| *Acrolocha diffusa* | 6 | 0 | 0 |  | 9 | 2 | 1 | | 18 |
| *Acrolocha helferi* | 0 | 0 | 0 |  | 4 | 0 | 0 | | 4 |
| *Anotylus sobrinus* | 1 | 0 | 1 |  | 3 | 0 | 0 | | 5 |
| *Anotylus tetracarinatus* | 1 | 0 | 0 |  | 0 | 0 | 0 | | 1 |
| *Arpedium cribatum* | 0 | 1 | 0 |  | 0 | 0 | 0 | | 1 |
| *Bisnius siegwaldii* | 0 | 0 | 0 |  | 5 | 1 | 0 | | 6 |
| *Bisnius tereus* | 0 | 2 | 0 |  | 0 | 0 | 0 | | 2 |
| *Bolitobius horni* | 12 | 8 | 7 |  | 11 | 6 | 12 | | 56 |
| *Bryophacis rubescens* | 0 | 1 | 0 |  | 1 | 0 | 0 | | 2 |
| *Bryoporus rufescens* | 1 | 0 | 0 |  | 1 | 1 | 0 | | 3 |
| *Deinopteroloma subcostatum* | 0 | 0 | 1 |  | 0 | 0 | 0 | | 1 |
| *Dinothenarus capitatus* | 6 | 3 | 0 |  | 3 | 1 | 0 | | 13 |
| *Dinothenarus pleuralis* | 141 | 117 | 97 |  | 63 | 76 | 77 | | 571 |
| *Eucnecosum brunnescens* | 0 | 61 | 45 |  | 120 | 57 | 306 | | 589 |
| *Eucnecosum tenue* | 1 | 0 | 6 |  | 2 | 2 | 3 | | 14 |
| *Eusphalerum pothos* | 5 | 0 | 2 |  | 4 | 0 | 0 | | 11 |
| *Gabrius brevipennis* | 26 | 6 | 6 |  | 32 | 26 | 23 | | 119 |
| *Habrocerus schwarzi* | 82 | 0 | 1 |  | 167 | 88 | 22 | | 360 |
| *Ischnosoma fimbriatum* | 8 | 13 | 17 |  | 14 | 12 | 9 | | 73 |
| *Ischnosoma pictum* | 0 | 0 | 0 |  | 1 | 0 | 0 | | 1 |
| *Ischnosoma splendidum* | 124 | 128 | 121 |  | 117 | 139 | 81 | | 710 |
| *Lathrobium fauveli* | 2 | 0 | 0 |  | 1 | 3 | 3 | | 9 |
| *Lathrobium washingtoni* | 5 | 2 | 3 |  | 4 | 2 | 1 | | 17 |
| *Lordithon fungicola* | 34 | 12 | 6 |  | 28 | 40 | 21 | | 141 |
| *Lordithon thoracicus thoracicus* | 2 | 0 | 0 |  | 3 | 1 | 0 | | 6 |
| *Megarthrus americanus* | 0 | 0 | 1 |  | 0 | 0 | 0 | | 1 |
| *Megarthrus angulicollis* | 13 | 12 | 22 |  | 12 | 4 | 5 | | 68 |
| *Megarthrus atratus* | 0 | 0 | 0 |  | 0 | 0 | 1 | | 1 |
| *Megarthrus excisus* | 5 | 0 | 0 |  | 18 | 6 | 4 | | 33 |
| *Micropeplus laticollis* | 18 | 91 | 77 |  | 168 | 221 | 58 | | 633 |
| *Mycetoporus americanus* | 50 | 32 | 29 |  | 21 | 83 | 77 | | 292 |
| *Mycetoporus nigrans* | 0 | 0 | 0 |  | 0 | 0 | 1 | | 1 |
| *Mycetoporus smetanai* | 0 | 1 | 3 |  | 0 | 1 | 1 | | 6 |
| *Nitidotachinus tachyporoides* | 2 | 2 | 27 |  | 11 | 1 | 6 | | 49 |
| *Olophrum consimile* | 0 | 1 | 0 |  | 0 | 1 | 3 | | 5 |
| *Olophrum rotundicolle* | 0 | 0 | 1 |  | 0 | 2 | 1 | | 4 |
| *Ontholestes cingulatus* | 2 | 0 | 0 |  | 0 | 0 | 0 | | 2 |
| *Oxyporus occipitalis* | 0 | 0 | 0 |  | 0 | 1 | 0 | | 1 |
| *Oxytelus fuscipennis* | 33 | 0 | 0 |  | 174 | 32 | 27 | | 266 |
| *Philonthus cyanipennis* | 1 | 0 | 0 |  | 0 | 0 | 0 | | 1 |
| *Philonthus fulcinius* | 1 | 0 | 0 |  | 2 | 0 | 0 | | 3 |
| *Phloeostiba lapponicus* | 1 | 1 | 0 |  | 0 | 0 | 0 | | 2 |
| *Proteinus limbatus* | 149 | 14 | 4 |  | 279 | 83 | 67 | | 596 |
| *Pselaphus bellax* | 0 | 0 | 0 |  | 0 | 0 | 1 | | 1 |
| **Supplementary Table S3**. *Continued.* | | | | | | | |  |  |
|  | **Mature** | | |  | **Regenerating** | | | | **Total** |
|  | **DD** | **MX** | **CD** |  | **DD** | **MX** | **CD** | |  |
| *Pseudopsis sagitta* | 79 | 65 | 54 |  | 67 | 127 | 116 | | 508 |
| *Pycnoglypta campbelli* | 2 | 0 | 0 |  | 0 | 0 | 0 | | 2 |
| *Quedius brunnipennis* | 9 | 63 | 28 |  | 26 | 26 | 13 | | 165 |
| *Quedius fellmani* | 6 | 7 | 4 |  | 6 | 3 | 2 | | 28 |
| *Quedius frigidus* | 4 | 2 | 7 |  | 3 | 4 | 3 | | 23 |
| *Quedius fulvicollis* | 39 | 16 | 11 |  | 37 | 20 | 27 | | 150 |
| *Quedius impar* | 6 | 3 | 5 |  | 23 | 13 | 3 | | 53 |
| *Quedius labradorensis* | 64 | 33 | 19 |  | 75 | 71 | 67 | | 329 |
| *Quedius molochinoides* | 0 | 0 | 6 |  | 0 | 5 | 11 | | 22 |
| *Quedius plagiatus* | 0 | 1 | 0 |  | 0 | 0 | 0 | | 1 |
| *Quedius rusticus* | 61 | 105 | 92 |  | 22 | 22 | 18 | | 320 |
| *Quedius simulator* | 3 | 2 | 17 |  | 25 | 13 | 10 | | 70 |
| *Quedius spelaeus* | 0 | 1 | 0 |  | 0 | 0 | 0 | | 1 |
| *Quedius uteanus* | 0 | 16 | 11 |  | 0 | 4 | 2 | | 33 |
| *Quedius velox* | 42 | 63 | 75 |  | 13 | 12 | 8 | | 213 |
| *Scaphium castaneipes* | 23 | 20 | 22 |  | 51 | 32 | 45 | | 193 |
| *Siagonium punctatum* | 0 | 0 | 0 |  | 0 | 0 | 3 | | 3 |
| *Stenus ageus* | 0 | 2 | 0 |  | 0 | 1 | 0 | | 3 |
| *Stenus austini* | 8 | 15 | 39 |  | 24 | 44 | 54 | | 184 |
| *Stenus mammops* | 4 | 1 | 2 |  | 9 | 10 | 20 | | 46 |
| *Stenus maritimus* | 1 | 0 | 0 |  | 1 | 1 | 3 | | 6 |
| *Stenus stygicus* | 0 | 0 | 1 |  | 2 | 4 | 3 | | 10 |
| *Tachinus basalis* | 1 | 0 | 0 |  | 4 | 2 | 0 | | 7 |
| *Tachinus elongatus* | 114 | 152 | 50 |  | 101 | 135 | 84 | | 636 |
| *Tachinus frigidus* | 100 | 180 | 87 |  | 134 | 87 | 56 | | 644 |
| *Tachinus fumipennis* | 418 | 25 | 17 |  | 183 | 38 | 15 | | 696 |
| *Tachinus luridus* | 1 | 0 | 0 |  | 0 | 0 | 0 | | 1 |
| *Tachinus quebecensis* | 5 | 6 | 1 |  | 4 | 4 | 3 | | 23 |
| *Tachinus vergatus* | 1 | 0 | 0 |  | 0 | 0 | 0 | | 1 |
| *Tachyporus abdominalis* | 0 | 0 | 0 |  | 4 | 0 | 0 | | 4 |
| *Tachyporus borealis* | 24 | 17 | 23 |  | 41 | 33 | 22 | | 160 |
| *Tachyporus canadensis* | 0 | 2 | 0 |  | 7 | 3 | 5 | | 17 |
| *Tachyporus chrysomelinus* | 0 | 0 | 0 |  | 1 | 0 | 0 | | 1 |
| *Tachyporus flavipennis* | 0 | 0 | 0 |  | 1 | 1 | 0 | | 2 |
| *Tachyporus maculicollis* | 0 | 0 | 0 |  | 2 | 0 | 0 | | 2 |
| *Tachyporus mexicanus* | 0 | 0 | 1 |  | 13 | 10 | 0 | | 24 |
| *Tachyporus nimbicola* | 0 | 0 | 0 |  | 1 | 0 | 0 | | 1 |
| *Tachyporus nitidulus* | 0 | 0 | 1 |  | 0 | 1 | 1 | | 3 |
| *Tachyporus pacificus* | 0 | 0 | 0 |  | 0 | 1 | 0 | | 1 |
| *Tachyporus rolomus* | 0 | 0 | 0 |  | 0 | 3 | 0 | | 3 |
| Aleocharinae | 1664 | 497 | 343 |  | 2602 | 1910 | 1243 | | 8259 |
| *Omalium* | 2 | 3 | 2 |  | 11 | 11 | 2 | | 31 |
| Other genera | 0 | 0 | 1 |  | 1 | 2 | 3 | | 7 |
| Number of individuals | 3511 | 1855 | 1433 |  | 5187 | 3773 | 2783 | | 18542 |
| Number of species | 52 | 45 | 46 |  | 58 | 59 | 52 | | 86 |
