# Supplementary material for: Natural succession and clearcutting as drivers of environmental heterogeneity and beta diversity in North American boreal forests
Source: PLoS One. 2018 Nov 2;13(11):e0206931. doi: 10.1371/journal.pone.0206931 (PMC6214561; doi:10.1371/journal.pone.0206931)
Supplement: S4 Table — (DOCX) [file pone.0206931.s004.docx]

| **S4. Table Carabids collected at deciduous-dominated (DD), mixed (MX) and coniferous-dominated (CD) mature and regenerating forests.** | | | | | | | | | |
| --- | --- | --- | --- | --- | --- | --- | --- | --- | --- |
|  | **Mature** | | |  | **Regenerating** | | | **Total** |  |
|  | DD | MX | **CD** |  | **DD** | **MX** | **CD** |  |  |
| *Agonum gratiossum* | 0 | 0 | 0 |  | 0 | 0 | 1 | 1 |  |
| *Agonum retractum* | 26 | 4 | 2 |  | 12 | 7 | 4 | 55 |  |
| *Agonum sordens* | 0 | 3 | 0 |  | 3 | 1 | 2 | 9 |  |
| *Amara littoralis* | 0 | 0 | 0 |  | 0 | 1 | 0 | 1 |  |
| *Calathus advena* | 2 | 86 | 43 |  | 6 | 4 | 5 | 146 |  |
| *Calathus ingratus* | 33 | 68 | 57 |  | 28 | 52 | 53 | 291 |  |
| *Calosoma frigidum* | 6 | 0 | 0 |  | 0 | 2 | 1 | 9 |  |
| *Carabus chamissonis* | 8 | 8 | 5 |  | 2 | 1 | 2 | 26 |  |
| *Carabus taedatus* | 0 | 1 | 2 |  | 0 | 1 | 0 | 4 |  |
| *Harpalus fulvilabris* | 1 | 0 | 0 |  | 2 | 3 | 3 | 9 |  |
| *Harpalus laticeps* | 0 | 0 | 0 |  | 0 | 0 | 1 | 1 |  |
| *Loricera pilicornis* | 0 | 0 | 0 |  | 1 | 0 | 0 | 1 |  |
| *Nebria gyllenhali* | 0 | 0 | 0 |  | 0 | 0 | 1 | 1 |  |
| *Patrobus foveocollis* | 53 | 8 | 8 |  | 31 | 21 | 20 | 141 |  |
| *Patrobus septentrionis* | 1 | 0 | 0 |  | 0 | 1 | 0 | 2 |  |
| *Platynus decentis* | 13 | 15 | 4 |  | 4 | 0 | 4 | 40 |  |
| *Pterostichus adstrictus* | 38 | 76 | 61 |  | 9 | 15 | 33 | 232 |  |
| *Pterostichus brevicornis* | 0 | 3 | 4 |  | 0 | 2 | 1 | 10 |  |
| *Pterostichus haematopus* | 4 | 71 | 92 |  | 2 | 7 | 12 | 188 |  |
| *Pterostichus pensylvanicus* | 8 | 13 | 13 |  | 0 | 5 | 7 | 46 |  |
| *Pterostichus punctatissimus* | 0 | 2 | 11 |  | 1 | 4 | 6 | 24 |  |
| *Synuchus impunctatus* | 9 | 0 | 2 |  | 13 | 31 | 40 | 95 |  |
| *Trechus apicalis* | 1 | 5 | 5 |  | 2 | 8 | 7 | 28 |  |
| *Trechus chalybeus* | 75 | 62 | 47 |  | 90 | 62 | 48 | 384 |  |
| Number of individuals | 278 | 425 | 356 |  | 206 | 228 | 251 | 1744 |  |
| Number of species | 15 | 15 | 15 |  | 15 | 19 | 20 | 24 |  |
