# Supplementary material for: Natural succession and clearcutting as drivers of environmental heterogeneity and beta diversity in North American boreal forests
Source: PLoS One. 2018 Nov 2;13(11):e0206931. doi: 10.1371/journal.pone.0206931 (PMC6214561; doi:10.1371/journal.pone.0206931)
Supplement: S5 Table — (DOCX) [file pone.0206931.s005.docx]

| **Table 1**. Environmental characteristics (mean ± SE) that did not differ significantly by cover type (deciduous-dominated (DD), mixed (MX) and conifer-dominated (CD) stands), stage (mature and regenerating forests) and their interaction. | | | | | | | | |
| --- | --- | --- | --- | --- | --- | --- | --- | --- |
|  | Mature | | | | Regenerating | | | |
|  | DD | MX | CD | DD | | MX | CD |  |
| Humus soil layer depth (cm) | 4.04 ± 0.57 | 4.36 ± 0.46 | 5.89 ± 0.65 | 4.85 ± 0.44 | | 5.40 ± 0.60 | 5.41 ± 0.47 |  |
| Fine woody debris cover (%) | 0.70 ± 0.18 | 1.72 ± 0.32 | 0.85 ± 0.18 | 0.59 ± 0.29 | | 0.54 ± 0.14 | 0.76 ± 0.25 |  |
| Coarse woody debris cover (%) | 6.96 ± 1.45 | 3.07 ± 1.23 | 4.41 ± 1.50 | 4.07 ± 1.19 | | 6.07 ± 1.92 | 5.44 ± 1.80 |  |
| Ground vascular plant cover (%) | 6.85 ± 1.09 | 13.22 ± 1.70 | 8.93 ± 1.47 | 3.52 ± 0.90 | | 4.33 ± 1.71 | 7.96 ± 1.35 |  |
| Lower vascular plant cover (%) | 22.98 ± 3.33 | 21.00 ± 2.54 | 16.89 ± 3.03 | 10.44 ± 1.65 | | 16.48 ± 2.36 | 20.31 ± 1.81 |  |
| Upper vegetation cover (%) | 14.56 ± 3.16 | 5.81 ± 2.27 | 7.59 ± 2.32 | 8.93 ± 1.45 | | 3.26 ± 1.52 | 2.96 ± 0.89 |  |
| Canopy openness (%) | 12.52 ± 0.94 | 10.71 ± 1.19 | 18.14 ± 2.18 | 8.87 ± 1.23 | | 17.35 ± 2.98 | 14.55 ± 1.57 |  |
| Tree diversity | 1.28 ± 0.08 | 1.77 ± 0.10 | 1.25 ± 0.08 | 1.65 ± 0.07 | | 1.39 ± 0.08 | 1.23 ± 0.08 |  |
